# Enhanced Heat-Powered Batteryless IIoT Architecture with NB-IoT for Predictive Maintenance in the Oil and Gas Industry

**DOI:** 10.3390/s25082590

**Published:** 2025-04-19

**Authors:** Raúl Aragonés, Joan Oliver, Carles Ferrer

**Affiliations:** 1 Department of Microelectronic and Electronic Systems, Universitat Autònoma de Barcelona, Bellaterra, 08193 Barcelona, Spain or raul.aragones@aeinnova.com (R.A.); joan.oliver@uab.cat (J.O.); 2 R&D Department, AEInnova—Alternative Energy Innovations, S.L., 08224 Barcelona, Spain

**Keywords:** energy harvesting, thermoelectricity, LCA, carbon footprint, NB-IoT, edge computing, NETZERO, energy-intensive industry

## Abstract

The carbon footprint associated with human activity, particularly from energy-intensive industries such as iron and steel, aluminium, cement, oil and gas, and petrochemicals, contributes significantly to global warming. These industries face unique challenges in achieving Industry 4.0 goals due to the widespread adoption of industrial Internet of Things (IIoT) technologies, which require reliable and efficient power solutions. Conventional wireless devices powered by lithium batteries have limitations, including a reduced lifespan in high-temperature environments, incompatibility with explosive atmospheres, and high maintenance costs. This paper proposes a novel approach to address these challenges by leveraging residual heat to power IIoT devices, eliminating the need for batteries and enabling autonomous operation. Based on the Seebeck effect, thermoelectric energy harvesters transduce waste heat from industrial surfaces, such as pipes or chimneys, into sufficient electrical energy to power IoT nodes for applications like the condition monitoring and predictive maintenance of rotating machinery. The methodology presented standardises the modelling and simulation of Waste Heat Recovery Systems (IoT-WHRSs), demonstrating their feasibility through statistical analysis of IoT-WHRS architectures. Furthermore, this technology has been successfully implemented in a petroleum refinery, where it benefits from the NB-IoT standard for long-range, robust, and secure communications, ensuring reliable data transmission in harsh industrial environments. The results highlight the potential of this solution to reduce costs, improve safety, and enhance efficiency in demanding industrial applications, making it a valuable tool for the energy transition.

## 1. Introduction

Climate change has become one of the most pressing challenges of our time, with global temperatures rising at an unprecedented rate. A significant contributor to this trend is the steady increase in waste heat generated by human activities, which intensifies the concentration of greenhouse gases, primarily carbon dioxide, in the atmosphere [1]. Since the mid-20th century, the industrial combustion of fossil fuels has been a major source of CO_2_ emissions, with large-scale industries responsible for a substantial portion of global energy losses. In Europe alone, these industries account for 21% of wasted energy, most of which is dissipated into the atmosphere as heat, representing over a quarter of the continent’s primary energy consumption [2].

To address these challenges, numerous green technologies are being developed to harvest energy from different physical domains and transform it into electrical energy [3,4], thereby helping to reduce the carbon footprint. Among these technologies, triboelectric and piezoelectric energy harvesting have shown significant potential in capturing mechanical energy from vibrations and motion, which can be harnessed to power low-energy devices in industrial settings [5]. Additionally, triboelectric nanogenerators (TENGs) and piezoelectric nanogenerators (PENGs) provide self-sustained power solutions for small-scale applications in environments where other forms of energy harvesting may not be as feasible.

Thermoelectric generators (TEGs) are commonly based on Bi2Te3 among other Seebeck effect materials [6] at the forefront of such technologies, harvesting heat to produce energy [7]. This dual benefit simultaneously generates electrical energy while reducing heat emissions into the atmosphere. Waste Heat Recovery Systems (WHRSs) [8,9] are a key example, transforming dissipated heat into electrical energy. These systems rely on the Seebeck effect, the transduction mechanism used in thermoelectric devices, typically Peltier cells. Based on the Carnot cycle for waste heat, WHRSs are generally designed to operate in sectors with medium-temperature differentials. For instance, the WHRS implemented at Bodegas Miguel Torres (HRS-Torres) [10] is used to harvest energy and monitor the status of a pellet stove.

While the energy output of thermoelectric devices is still insufficient to power systems with high energy demands, they can generate enough energy to sustain wireless IoT devices. This makes IoT applications one of the primary use cases where TEG devices can provide sufficient energy to power systems [3,11,12]. Many IoT nodes today rely on batteries, which are not only expensive and environmentally harmful but also pose significant limitations in industrial environments. In contrast, WHRSs can harvest energy from any hot surface, enabling self-powered IoT nodes in remote or hard-to-reach areas of industrial facilities, without requiring electrical power distribution. Furthermore, WHRSs can operate in hazardous environments where batteries or grid power would pose safety risks.

Recent advancements in energy-harvesting technologies, such as wireless power harvesting, have also contributed to the growing trend of self-powered systems. In particular, backscatter communication systems that harvest energy from ambient wireless signals represent an emerging approach in energy-efficient IoT devices [13].

This paper expands on this concept by incorporating the benefits of Narrowband IoT (NB-IoT) technology, a key enabler for robust and secure long-range wireless communications in industrial settings. NB-IoT eliminates the need for extensive wiring, significantly reducing installation costs and complexity in large facilities. Its robustness and security make it particularly well suited for harsh industrial environments, including explosive or high-temperature areas. For example, in a petroleum refinery, the integration of WHRS-powered IoT nodes with NB-IoT technology has demonstrated a reliable and efficient solution for condition monitoring and predictive maintenance, even in challenging operational conditions. Additionally, this protocol will allow the implementation of spectrum analysis for electric machine predictive maintenance standard implementation (ISO 10816 [14]).

Building on prior work, this paper presents an improved architecture for Waste Heat Recovery Systems (IoT-WHRSs), designed to power IoT nodes. This new architecture enhances the performance and efficiency of earlier designs by addressing key limitations, particularly those related to thermal path optimisation and air gaps within the system enclosure. The complete thermoelectric system model includes the contribution of the TEG and all other components of the WHRS. A dynamic model for the thermoelectric device is employed, which captures its behaviour in transforming heat power into electrical energy [15]. However, the physical parameters of TEG cells, such as the Seebeck coefficient, thermal conductivity, and thermal resistivity, are often not provided by manufacturers and must be calculated using datasheet figures [16]. Effective parameter calculation methods are applied in this study [17].

The WHRS design also incorporates thermal resistances and capacitances for all elements along the thermal path, including contact resistances and heatsink fin behaviour, which significantly affect system efficiency [18,19]. Within this context, this paper outlines a methodology for modelling and simulating the improved IoT-WHRS architecture. The experimental validation demonstrates that the enhancements achieve superior efficiency and better performance compared to the earlier design.

The paper is structured as follows: Section 2 introduces the key benefits of predictive maintenance for Industry 4.0. The architecture of the improved IoT-WHRS for IoT applications, the modelling methodology of the IoT-WHRS, and the finite difference model are detailed in Section 3. The detailed hardware architecture is introduced in Section 4. Simulation and experimental results, as well as the final installation of the pilot in the CEPSA oil refinery, are discussed in Section 5. Section 6 presents the background and related work. Finally, the Conclusions summarise this work and discuss future opportunities for IoT-WHRSs.

## 2. Industrial IoT Application for Predictive Maintenance

### 2.1. Main Benefits

**Figure 1:**
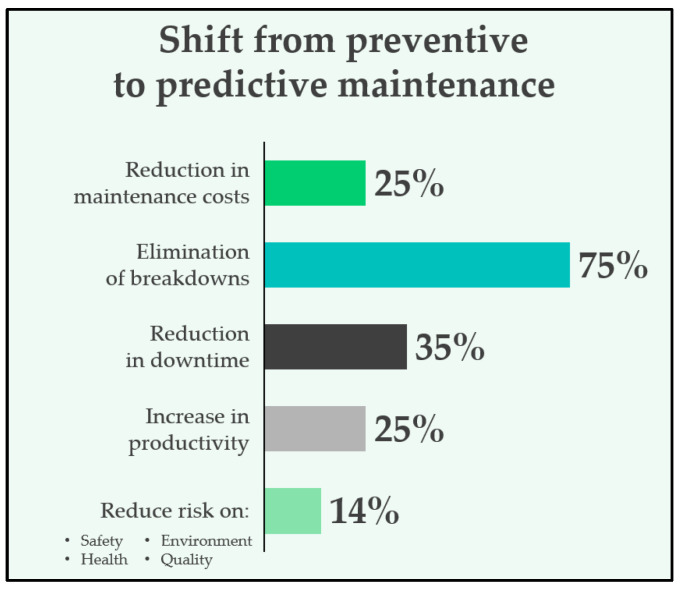
Benefits of predictive maintenance versus preventive maintenance.

Predictive maintenance has revolutionised the operation of electric machines by enabling the early detection of potential failures, optimising maintenance schedules and minimising unplanned downtime. One of its greatest advantages lies in its ability to extend machine life while reducing operational costs. In recent years, integrating predictive maintenance with IoT devices has further enhanced its effectiveness, particularly in industrial environments where energy efficiency and real-time data processing are critical. The benefits for industries are illustrated in Figure 1.

### 2.2. Velocity Measurement

A key component of predictive maintenance is vibration monitoring, which serves as a reliable indicator of a machine’s health. Standards like ISO 10816 provide clear guidelines for evaluating the mechanical condition of rotating machinery by measuring vibration velocity in root mean square (RMS) values (mm/s). The standard categorises the machine condition into three distinct zones:

- Green Zone: Normal operation, no intervention needed;
- Yellow Zone: Warning level, maintenance should be scheduled;
- Red Zone: Critical level, immediate action required to prevent failure.

**Figure 2:**
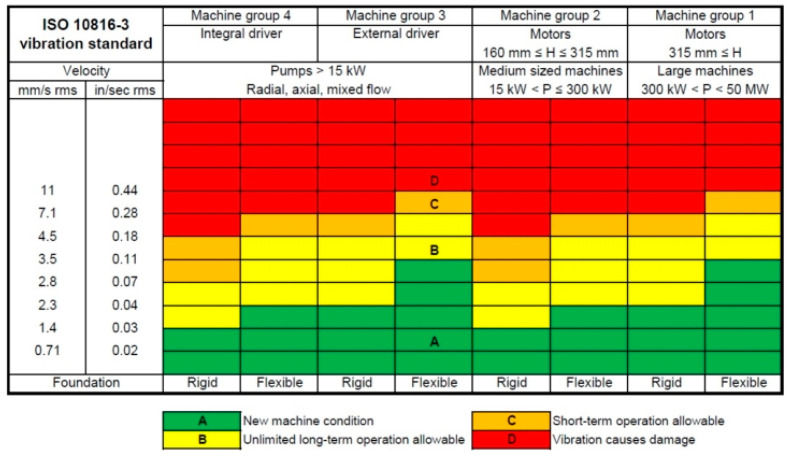
Colour-coded threshold for a pump, applying ISO 10816 [20].

These thresholds simplify the assessment of machine health and are commonly represented in predictive maintenance tools using a colour-coded system (Figure 2), allowing maintenance teams to quickly interpret data and act proactively. By combining IoT-enabled devices with vibration-monitoring standards like ISO 10816, industrial environments can achieve safer, more efficient, and cost-effective maintenance strategies.

### 2.3. Spectral Analysis for Fault Diagnosis

While RMS vibration velocity (mm/s) provides an excellent overall indicator of machine health, incorporating spectral analysis offers a much deeper level of fault diagnosis. By analysing vibration frequencies in the spectrum, it becomes possible to identify specific anomalies in the motor and determine their cause. This capability transforms the device into a powerful diagnostic tool, enabling targeted maintenance actions during scheduled downtimes, minimising operational interruptions, and preventing catastrophic failures.

**Figure 3:**
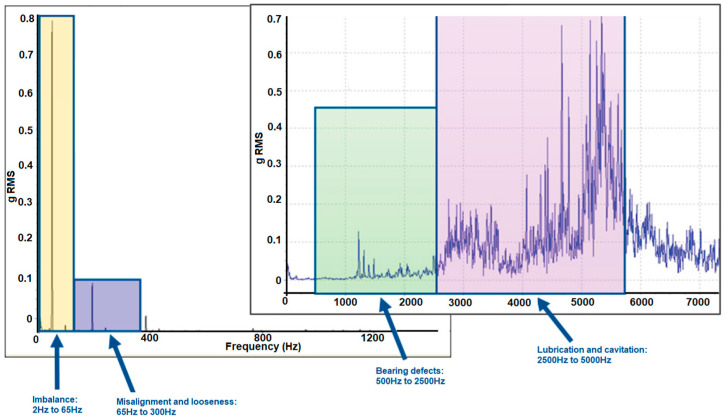
Analysis spectrum of vibrations to determine potential malfunctions.

A vibration frequency analysis in Hertz (Hz) can reveal the signature patterns of various types of faults (Figure 3):

- Imbalance: Detected at the rotational frequency of the motor (1× RPM or the fundamental frequency, typically from 2 Ht to 65 Hz);
- Misalignment: Typically appears at 1× RPM and higher harmonics (e.g., 2 × RPM, 3 × RPM typically from 65 Hz to 300 Hz);
- Bearing faults: Characterised by specific frequencies such as the Ball Pass Frequency Outer (BPFO), Ball Pass Frequency Inner (BPFI), or fundamental train frequency (FTF), which depend on the bearing geometry and rotational speed, typically from 500 Hz to 2500 Hz;
- Lubrication and cavitation issues: Detects a lack of lubrication in the gearboxes, as well as air bubbles in the lubrication, typically from 2500 Hz to 5000 Hz.

By monitoring these specific frequencies, maintenance teams can pinpoint the origin of problems, such as an unbalanced rotor, misaligned shafts, or damaged bearings, allowing for swift corrective actions. The ability to measure and analyse vibration in both RMS velocity and spectral domains provides a comprehensive toolkit for ensuring the reliability and efficiency of industrial machinery.

## 3. Heat-Powered IIoT Architecture

### 3.1. Introduction to IoT-WHRS Architecture

A low power consumption is a key requirement for IoT nodes deployed in predictive maintenance applications. These nodes often operate in environments with limited energy availability and experience bursts of high energy demand during data transmission. Thermoelectric energy-harvesting systems (IoT-WHRSs) have emerged as a sustainable solution for powering IoT nodes in such scenarios. These systems harvest energy from waste heat in facilities, storing it in energy buffers for efficient use during data acquisition, processing, and communication cycles.

**Figure 4:**
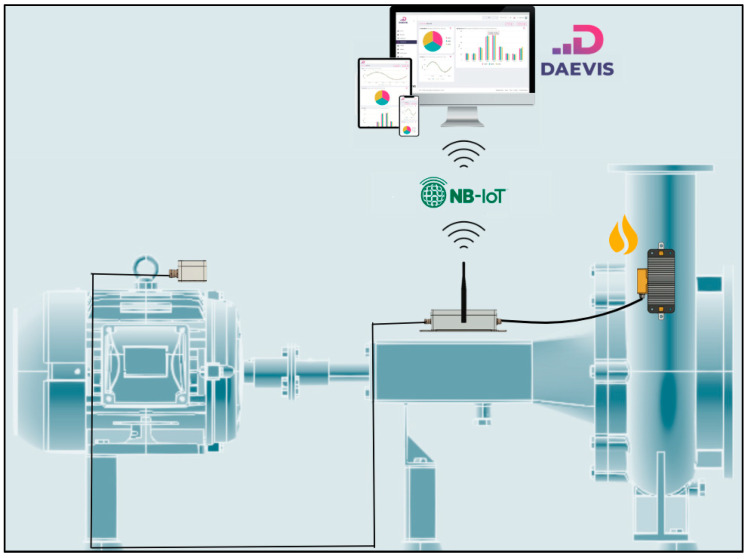
Energy-harvesting architecture in IIoT nodes.

Figure 4 illustrates the architecture of a wireless IoT node powered by an energy-harvesting system. The data acquisition collects information through smart sensors installed over the electric machine (left), processes it, and transmits it using an NB-IoT communication protocol (centre). Meanwhile, the energy-harvesting device (right) consists of components that harvest, rectify, regulate, and store the harvested energy, ensuring the node is powered only when necessary. Finally, all data are represented in a visualiser, typically in a cloud platform (top). In our case, this cloud platform is named DAEVIS (Dynamic AEInnova Visualiser) running on AWS. AEInnova is a company founded by the authors 10 years ago.

### 3.2. IoT-WHRS Model Parametrisation

Thermoelectric energy-harvesting systems must overcome several challenges to achieve a high efficiency. Maintaining a significant temperature difference between the hot and cold sides of the thermoelectric generator (TEG) is essential for maximising power output. The efficiency of the IoT-WHRS is influenced by the thermal resistance between the TEG, heat collector, and heatsink, as well as the presence of air gaps created by clamping mechanisms. These air gaps act as resistive thermal paths, reducing the overall system efficiency.

**Figure 5:**
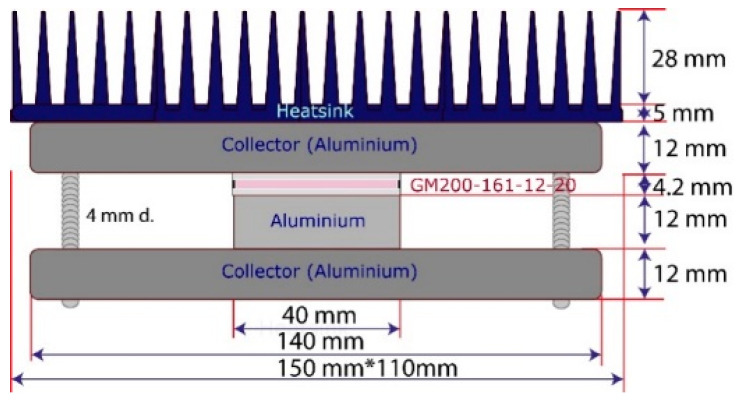
IoT-WHRS system and dimensions.

Figure 5 presents the IoT-WHRS architecture used in this study, comprising the following:

- A GM200-161-12-20 Peltier cell acts as the TEG;
- An aluminium plate serves as the heat transmitter between the aluminium collector and the TEG;
- Finally, the system is fastened between two pieces of aluminium Al6063-T5, corresponding to the collector and the base of the heatsink, which are attached to an Al6063-T5 heatsink base. This optimised design allows the IoT-WHRS to harness energy efficiently from waste heat.

The power generation of the IoT-WHRS (Waste Heat Recovery System) is determined by the thermoelectric generator (TEG) and depends on the system’s architecture and materials. The thermal performance is modelled through thermal resistances and capacities, influenced by the physical properties of the materials used, such as density, thermal conductivity, and specific heat, which are summarised in Table 1.

Key factors include the following:

- Thermal Properties of Materials: Materials like copper, aluminium alloys (Al6063-T5), alumina, graphite, and mica contribute differently to heat transfer. Copper offers the highest thermal conductivity, while air is modelled as an ideal gas due to its low density;
- Contact Resistance: Microhardness and surface roughness influence the contact resistance between materials, affecting heat transfer efficiency [21]. Air gaps caused by surface irregularities can increase resistance, reducing performance;
- TEG Model: The TEG’s performance is modelled using an “effective parameter model” due to limited manufacturer specifications, incorporating properties of its pellets, ceramics, and welds;
- Heatsink Efficiency: The heatsink’s fins dissipate heat to the environment, while the base transfers heat from the TEG. The material and geometry of the fins are crucial for cooling performance [22].

In summary, the IoT-WHRS system’s efficiency depends on minimising thermal resistance through material optimisation and precise control of contact interfaces, ensuring effective heat capture and dissipation.

### 3.3. Thermal Modelling of IoT-WHRS

**Figure 6:**
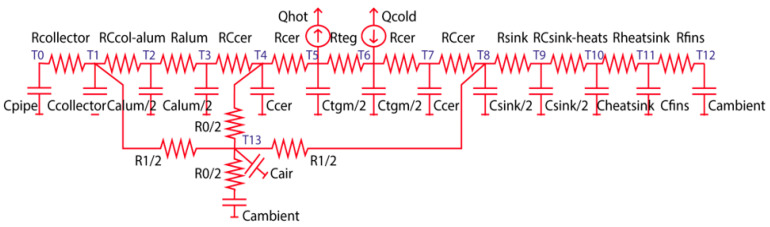
Thermal resistances and heat capacitances of the model.

Figure 6 illustrates the thermal resistances and capacitances that play a central role in the IoT-WHRS model. Each component in the system contributes to heat transfer by acting as a thermally resistive element.

The heat storage capacity of the materials is determined by their specific heat, while the thermal resistance and capacitance of the air gap are integral to the system’s overall performance. The IoT-WHRS establishes two distinct heat transfer pathways: one through the elements surrounding the TEG and another across the air channel. Key contributors to heat flow and distribution include the following:

- Collector and heatsinks: Constructed from aluminium to optimise heat conduction;
- Thermoelectric generator (TEG): Encompasses the thermoelectric effect, ceramic plates, and welds connecting these plates. Heat rejection and absorption are represented by Q_hot_ and Q_cold_, respectively;
- Heatsink and its fins: Responsible for dissipating heat into the surrounding environment;
- Contact interfaces: Points of interaction between components introduce thermal resistance that impedes heat transfer;
- Airgap volume: Acts as a thermal barrier, contributing to the overall resistance within the system.

Additionally, the thermal circuit accounts for the TEG’s dynamic behaviour to refine the model’s accuracy. The heat transfer mechanism in the IoT-WHRS is characterised by two distinct channels, as previously outlined, which work together to manage the heat flow efficiently.

#### 3.3.1. Heat Transfer Contribution via the TEG Channel

Heat transfer within the TEG channel involves contributions from multiple components. Following the models described in [13], the TEG’s thermal behaviour accounts for the resistive and capacitive properties of its thermoelectric pellets, represented as R_teg_ and C_teg_. These parameters are temperature-dependent and must be recalculated dynamically during simulations. Surrounding the pellets are ceramic plates (made of alumina), which add their own thermal resistance (R_ceramics_) and heat capacity (C_ceramics_), derived from the material’s conductivity, heat capacity, and geometry.

The interconnecting aluminium between the pellets is excluded from direct modelling because the effective parameter approach accounts for its contribution, as explained in Section 4.

The aluminium collector is modelled using its thermal resistance (R_collector_) and heat capacity (C_collector_), while the aluminium separator plate (positioned between the collector and the TEG) is represented by R_alum_ and C_alum_.

The heatsink requires a more intricate model. Its base and fins are treated as separate components. The base, similar to the collector plate, is modelled with R_heatsink_ and C_heatsink_. Meanwhile, the fins, critical for heat dissipation, are represented by R_fins_ and C_fins_ as per the approach in [19].

Heat flow originates in the hot surface, where it is collected by the aluminium plate, and it dissipates into the environment via the heatsink. The machine’s hot surface (where we are harvesting the heat) and the external environment are modelled as high-capacity thermal reservoirs (C_pipe_ and C_ambient_), effectively considered infinite in the simulation.

All RC_xxx_ resistances in the thermal circuit account for contact resistances, which are critical factors for accurate heat transfer modelling, as detailed in [18].

#### 3.3.2. Heat Transfer Modeling via the TEG Channel

The airgap channel represents the air layer formed by the clamping mechanism that secures the TEG between the collector and the heatsink. This enclosed air volume significantly impacts the system’s thermal performance by contributing to heat flow around the TEG. The air layer is confined by the collector and the aluminium heatsink base, which has a high thermal resistivity.

To capture the thermal effects of the airgap, the model incorporates several thermal resistances and capacities:

- R_0_: Represents the transverse heat transfer between the TEG and the surrounding environment;
- R_1_: Models the thermal channel connecting the heatsink and the collector;
- R_2_: Accounts for heat transfer between the insulator and the collector;
- C_air1_ and C_air2_: Represent the heat storage capacities of the airgap channels.

This layered modelling approach ensures a more precise representation of heat propagation within the airgap and its interaction with the overall system.

### 3.4. TEG Effective Parameters Model

The efficiency of the IoT-WHRS system is strongly tied to the performance of the thermoelectric module, which converts thermal energy into electrical energy. The energy output of the TEG is driven by the interplay of key physical phenomena within the thermoelectric semiconductors, the Seebeck, Peltier, Thomson, and Joule effects, as summarised in Equations (1)–(6). To enhance energy production, the Seebeck effect must be maximised while minimising heat transfer. Heat transfer within the TEG is predominantly influenced by its internal resistance, R_0_, which impacts the overall efficiency, denoted by the maximum efficiency coefficient (η_max_). The figure of merit (Z_T_) is used to benchmark thermoelectric performance.

(1) $$\mathrm{Seebeck}\;\mathrm{effect}:\;\frac{\partial E_{T}}{\partial T}=\alpha_{a}-\alpha_{a}$$

(2) $$\mathrm{Peltier}\;\mathrm{effect}:\;{\dot{Q}}_{Peltier}=\pm IT\left(\alpha_{a}-\alpha_{b}\right)$$

(3) $$\mathrm{Thomson}\;\mathrm{effect}:\;{\dot{Q}}_{Thomson}=-\sigma I∆T$$

(4) $$\mathrm{Joule}\;\mathrm{effect}:\;{\dot{Q}}_{Joule}=R_{0}I$$

(5)             Efficiency coefficient:                        ηmax=TH−TCTH1−ZT¯−11+ZT¯−TCTH

(6) $$\;\mathrm{Figure}\;\mathrm{of}\;\mathrm{merit}:\;ZT=\frac{\sigma \alpha^{2}T}{\kappa }$$

Note: E, $\dot{Q}$, T. I, α, σ, κ, and R_0_ mean electromotive force (V), heat flow (W), temperature (K), current (A), Seebeck coefficient (V/K), electrical conductivity (S/m), thermal conductivity (W/(mK)), and electrical resistance (Ω) respectively. Figure of Z-merit units are 1/K.

To simulate the TEG’s behaviour in the IoT-WHRS system, the thermoelectric properties—Seebeck coefficient (α), electrical conductivity (σ), and thermal conductivity (κ)—must be determined. However, these parameters are rarely provided by manufacturers. Instead, the model employs effective parameters (α^∗^, κ^∗^, ρ_∗_, and ZT_∗_) derived from the TEG’s datasheet specifications. This approach introduces minimal errors while providing sufficient accuracy for simulation purposes [23]. As shown in Equations (7) and (8), the effective parameters are calculated using maximum power (W_max_), open-circuit voltage (V_max_), maximum current (I_max_), and efficiency (η_max_).

(7) $$\alpha^{*}=\frac{4W_{max}}{nI_{max}\left(T_{h}-T_{c}\right)}r^{*}=\frac{4{\left(\frac{A}{L}\right)W}_{max}}{n{\left(I_{max}\right)}^{2}}$$

(8) $$k^{*}=\frac{{\left(\alpha^{*}\right)}^{2}}{\rho^{*}Z^{*}}\;Z^{*}=\frac{2}{T_{c}\left(1+{\left(\frac{T_{c}}{T_{h}}\right)}^{-1}\right)}\left[{\left(\frac{1+\left(\frac{{ƞ}_{max}}{{ƞ}_{c}}\right)\left(\frac{T_{c}}{T_{h}}\right)}{1-\left(\frac{{ƞ}_{max}}{{ƞ}_{c}}\right)}\right)}^{2}-1\right]$$

Note: η_c_ = 1 − TH/TC is the Carnot efficiency.

The number of thermocouples in the TEG module (n) is also incorporated. This study utilises the Kryotherm GM200-161-12-20 module. Table 2 presents its key specifications and the corresponding effective parameters calculated based on Equations (7) and (8).

### 3.5. IoT-WHRS Finite Difference Model

The IoT-WHRS simulation focuses on capturing the thermal energy flow from the hot pipe temperature to the cooler ambient temperature, with the thermoelectric module converting the captured heat into electrical energy. The system is modelled under the constraint that the electrical power generated is equal to the thermal energy difference between the hot (Q_H_) and cold (Q_C_) heat fluxes.

The heat transfer within the system is described by a set of partial differential equations that characterise the temperature distribution across the components. As heat generation in the TEG varies with temperature, the system exhibits non-linear behaviour. Solving these non-linear equations analytically is impractical, so the system is discretised using finite difference equations [19]. Starting with the initial pipe (T_pipe_) and ambient (T_ambient_) temperatures, the system’s internal temperature evolution is computed iteratively.

The thermoelectric module’s power output is determined by the cold-side temperature of the TEG and the temperature gradient across its surfaces. This numerical approach transforms the heat transfer equations into a discrete set of difference equations, as expressed in Equation (9). This iterative methodology allows for the simulation of temperature dynamics and power generation under varying conditions.

(9) $$\left(1+\frac{\delta t}{C_{a}}\sum_{b=1}^{n}\frac{1}{R_{a,b}}\right){T^{\prime }}_{a}-\frac{\delta t}{C_{a}}\sum_{b=1}^{n}\frac{1}{R_{a,b}}{T^{\prime }}_{b}=T_{a}+\dot{Q_{a}}\frac{\delta t}{C_{a}}$$

The subscripts *a* and *b* correspond to adjacent nodes connected through the thermal resistance R_a,b_. In this formulation (as shown in the models of Figure 6), T represents the current temperature at a node, while T′ is the temperature to be determined in the current time step (δt) iteration. R_a,b_ denotes the thermal resistance between the two nodes, C_a_ is the thermal capacity assigned to the node, and Q_a_˙ represents the heat generated at the node.

The matrix terms (depicted in Equation (9) by the expression in parentheses) are derived based on the thermal resistance and thermal capacity values defined by the model connections. For instance, in the IoT-WHRS model (Figure 6), there are 14 nodes, leading to the creation of a 14 × 14 matrix, as shown in Equation (10).

(10) $$\left(\begin{array}{ccc} \begin{array}{ccc} m00 & m01 & 0\\ m10 & m11 & m12\\ 0 & m21 & m22 \end{array} & \cdots & \begin{array}{ccc} 0 & 0 & 0\\ 0 & 0 & m1d\\ 0 & 0 & 0 \end{array}\\ \vdots & \ddots & \vdots \\ \begin{array}{ccc} 0 & 0 & 0\\ 0 & 0 & 0\\ 0 & md1 & 0 \end{array} & \cdots & \begin{array}{ccc} mbb & mbc & 0\\ mcb & mcc & mcd\\ mdb & mdc & mdd \end{array} \end{array}\right)\;$$

Each entry in the matrix is determined by the connections defined in the model. For instance, the non-zero elements in the first two and last two rows of this matrix are calculated based on the thermal links shown in Figure 6. These terms are derived directly from the relationships and interactions outlined in the figure.

*                                            m00 = 1 + (dt/C_pipe_) (1/R_col_)*

*                                         m01 = −(dt/C_pipe_) (1/R_col_)*

*                                       m10 = −(dt/C_col_) (1/R_col_)*

*                                                                              m11 = 1 + (dt/C_col_) (1/R_col_ + 1/R_ccolalum_ + 1/R_air1_)*

*                                              m12 = −(dt/C_col_) (1/R_ccolalum_)*

*                                        m1d = −(dt/C_col_) (1/R_air1_)*

*…*

*                                         mba = −(dt/C_fins_) (1/R_dis_)*

*                                                             mbb = 1 + (dt/C_fins_) (1/ R_dis_ + 1/R_fins_)*

*                                          mbc = −(dt/ C_fins_) (1/R_fins_)*

*                                          mcb = −(dt/C_amb_) (1/R_fins_)*

*                                                             mcc = 1 + (dt/C_amb_) (1/R_fins_ + 1/R_air0_)*

*                                            mcd = −(dt/C_amb_) (1/R_air0_)*

In this equation, several terms depend on the temperature and must be recalculated at each simulation step. During each iteration, the finite difference system solves for the current temperatures, requiring the inversion of the state matrix—a computationally intensive task. Additionally, all temperature-dependent parameters must be updated at every step, including the thermal resistance of the fins, the resistances and capacities of the airgap, the thermal resistance of the contacts, and the heat transfer within the TEG.

## 4. Edge Computing for Batteryless Device with NB-IoT

This section outlines the development of an innovative wireless IIoT edge device powered by energy harvesting, which operates without traditional batteries. Instead, the system employs supercapacitors as the primary energy storage component. These devices support up to one million charge/discharge cycles, offer a faster energy transfer, and eliminate the use of critical raw materials such as lithium and cobalt. By integrating NB-IoT communication and real-time edge-computing capabilities, the device overcomes key limitations in industrial monitoring and predictive maintenance applications, while maintaining a sustainable and maintenance-free design.

### 4.1. Eliminating Batteries: A New Paradigm for IIoT Monitoring

IIoT devices typically require 30–150 mW of power, which is traditionally supplied by lithium batteries. However, batteries face significant challenges in industrial environments, especially in compact devices where limited space reduces energy storage capacity, shortening the operational lifespan of IoT nodes to just a few months or years depending on data acquisition frequency. This leads to recurring costs for replacement and maintenance, logistical difficulties, and environmental concerns. These issues are aggravated in large-scale industrial facilities and hazardous environments (e.g., EX/ATEX zones), where lithium batteries are prohibited due to safety regulations. As a result, these limitations have historically impeded the adoption of wireless IIoT technology for critical applications like vibration monitoring and predictive maintenance.

To address these challenges, the thermoelectric energy-harvesting device developed in this work has been sized for powering high-energy sensors and advanced computing processes without relying on batteries. Thermoelectric generators (TEGs) provide a continuous and sustainable power supply, enabling high-frequency data acquisition, real-time edge computing, and long-range wireless communication.

### 4.2. Overcoming Technological Barriers for Wireless IIoT

Industrial monitoring systems often require real-time data acquisition and processing, secure communication protocols, and a minimal reliance on third-party systems. These demands place significant constraints on battery-powered IoT devices, which are further affected by the battery storage capacity (mAh), the environmental conditions, such as temperature and humidity, and component power consumption, including the following:

- Efficiency of DC/DC converters;
- Processor operations, such as the following:
  ▪ Operational frequency (MHz);
  ▪ Power-saving modes (e.g., sleep, ultra-sleep);
  ▪ Edge-computing algorithms and firmware optimisation;
- Sensor power requirements and signal conditioning electronics;
- Wireless communication protocol energy usage.

By leveraging energy harvesting, the proposed IIoT device overcomes these barriers, supporting the following:

- Edge-computing capabilities for advanced data processing, including FFT, filtering, and ML/DL algorithms;
- Continuous operation in hazardous environments (ATEX) without requiring battery replacements;
- Data transmission at high throughput rates of up to 256 Kbps.

### 4.3. NB-IoT: The Optimal Protocol for Industrial Applications

Selecting the appropriate wireless protocol is a critical decision for industrial wireless sensor networks, as it directly affects node energy consumption, communication range, network scalability, and security. For edge-computing IIoT devices, the protocol must also support robust communication in challenging industrial environments while maintaining a low latency and high data throughput.

Among Low-Power Wide-Area Network (LPWAN) protocols, Narrowband IoT (NB-IoT) stands out as an ideal solution for energy-efficient, long-range communication. According to studies by 3GPP, GSMA, and Kais et al. [24], NB-IoT offers significant advantages for industrial applications, including scalability (large numbers of devices can connect to a single gateway), low latency (it ensures real-time monitoring and data transmission), data security (encrypted communication for critical applications), and cost efficiency (it operates on licensed frequency bands, ensuring reliability and reduced interference).

In this work, NB-IoT is integrated into the energy-harvesting IIoT device to provide a secure and efficient communication protocol for industrial applications. For example, in the oil and gas sector, the system has been successfully deployed for vibration monitoring and predictive maintenance in high-risk environments. The integration of NB-IoT enables robust and reliable data transmission across large facilities, reducing infrastructure costs and enhancing operational safety.

### 4.4. Internal Architecture of the Edge-Computing Node

**Figure 7:**
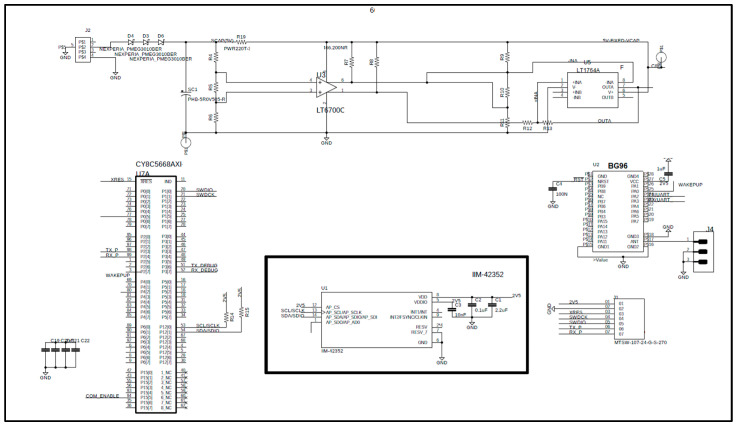
IIot board with the processing and communicating sub-circuits.

**Figure 8:**
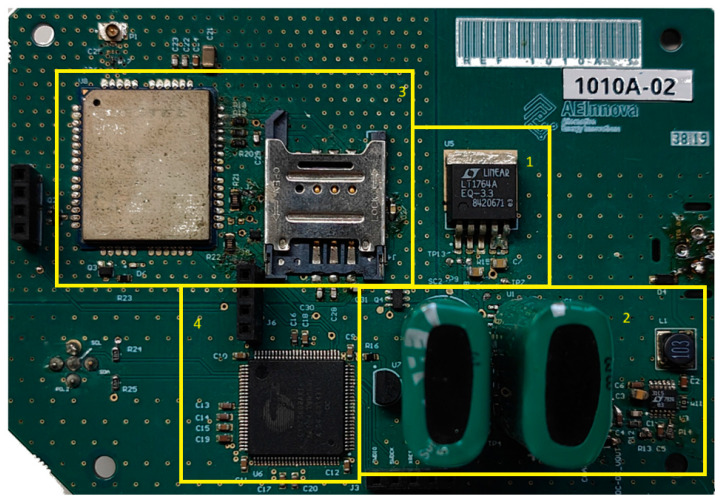
PCB with DC/DC MPPT (1), supercapacitor (2), communication (3) and processing (4) elements.

Figure 7 and Figure 8 show the internal architecture of the NB-IoT sensing node, which is composed of the following key components:

- Communications Hardware: Includes a 5 dBi antenna, the Quectel BG96 NB-IoT UART module, and a SIM card for wireless connectivity (from Vodafone);
- Power Electronics: Comprises a DC/DC converter with MPPT to supply power to the external sensor, an energy buffer for alternative energy sources, energy management circuitry, and an SPI bus interface connected to an internal three-axis vibration IMU (Inertial Measurement Unit) from STMicroelectronics;
- Programmable Cortex M3 (32-bit processor), integrated Flash and RAM memory, analogue and digital FPGA capabilities, and communication interfaces such as digital communication buses.

This modular architecture enables efficient sensing, processing, and communication for industrial IoT applications.

### 4.5. Data Acquisition, Processing, and Communication Flow

To calculate data latency and size the energy buffer, it is essential to first determine the total energy harvested to meet the system’s energy requirements. Data latency depends on the power generated by the thermoelectric unit and the energy consumed by the edge-computing algorithm, assuming up to one data package is transmitted per minute at 150 °C.

The process is divided into five time slots or operational states, each with a specific duration and power consumption. These factors collectively determine the energy cost of each state is detailed in Section 5.2.

## 5. Results

### 5.1. WHRU Characterisation

#### 5.1.1. Simulation Results

Table 3 presents the setup parameters for the IoT-WHRS simulation, which correspond to the configuration of the devices under test. R_load_ represents the external electrical resistance coupled to the system, matching the internal electrical resistance of the thermoelectric device to achieve a maximum power output. Torque, force, and screw diameter define the mechanical fixing parameters that secure the thermoelectric device to the collector and heatsink.

The model parameters of the IoT-WHRS system are derived from the physical characteristics of the components used (Figure 5) and the material properties listed in Table 1. Contact resistances and fin dissipation capacities are calculated based on references [15,16], respectively.

**Figure 9:**
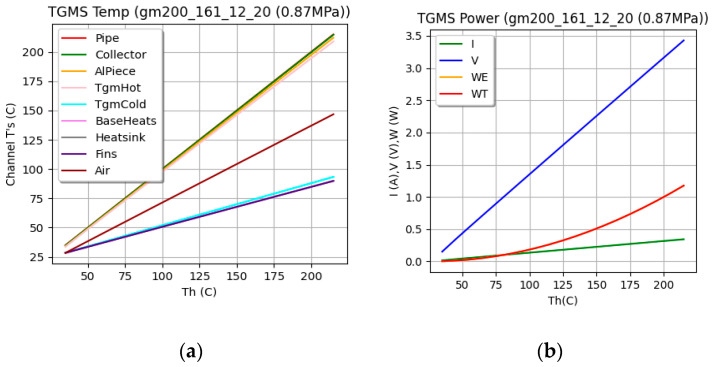
(**a**) Results of the IoT-WHRs simulation, (**b**) power supplied by the system.

Figure 9 shows the results of a simulation of the IoT-WHRS. The left figure (a) illustrates the temperature evolution, and the right (b) depicts the power output of the systems. The simulation spans a Thot range from 25 °C to 225 °C, while Tcold is maintained at 25 °C. As expected, the thermal power harvested (WT) matches the electrical power generated (WE). The power output of the two IoT-WHRS architectures is comparable.

#### 5.1.2. Experimental Results

The device was tested across a temperature range from room temperature to 200 °C. The temperature and power evolution of the system were recorded using temperature sensors placed inside the module. The parameters were maintained as specified in the simulation (Table 3).

**Figure 10:**
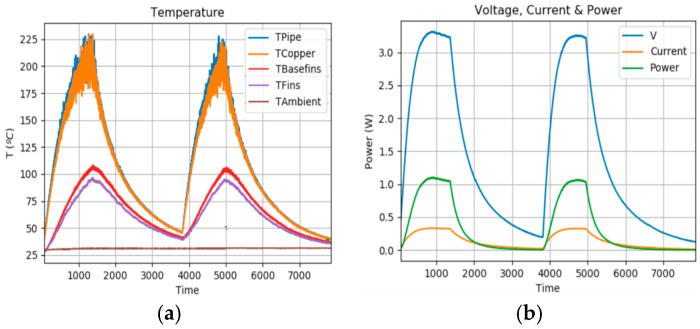
Temperature evolution (**a**) and power generated (**b**) results.

The test was conducted by heating the collector of the IoT-WHRS system. During each experiment, the collector was heated until its temperature stabilised at approximately 200 °C (Figure 10), after which it was allowed to cool back to room temperature. Five heating and cooling cycles were performed during each experiment. On average, one hour was required for the heating phase and another hour for the cooling phase. Temperature measurements were taken every second at specific locations within the system. In the graphs, Tpipe represents the temperature of the collector, Tcopper corresponds to the copper separator, Theatsink represents the base of the heatsink, Tbasefins is the temperature at the base of the heatsink fins, and Tfins refers to the tip of the fins. Additionally, the room temperature was also measured.

The left plot illustrates the temperature variations during the heating and cooling cycles. At higher temperatures, the data show greater dispersion due to the reduced sensitivity of thermistors in this range. The right plot displays the measured voltage, current, and power.

The experimental results align closely with the simulation outcomes. Similarly, the system’s power behaviour during the heating and cooling cycles is consistent. This is because the power generated depends on the temperature of the hot side and the temperature difference across the TEG.

#### 5.1.3. Statistical Results

**Figure 11:**
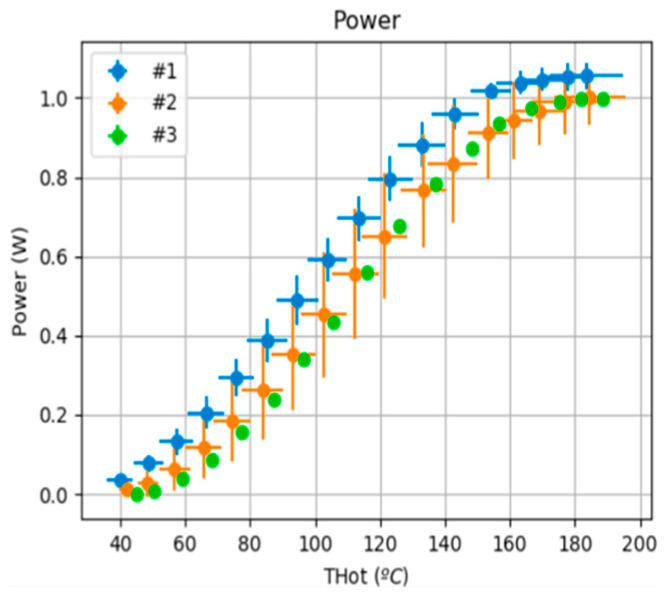
Power graph of the IoT-WHRS.

Several experiments were performed using three GM200-161-12-20 TEG cells to analyse the behaviour of the IoT-WHR systems (Figure 11). A total of 21 tests with heating and cooling cycles were performed. The points represent the mean value and the crosses the variability. The figure shows the good behaviour of the cells in each IoT-WHRS. The power generated is similar for each thermoelectric cell, remaining inside the error bar.

**Figure 12:**
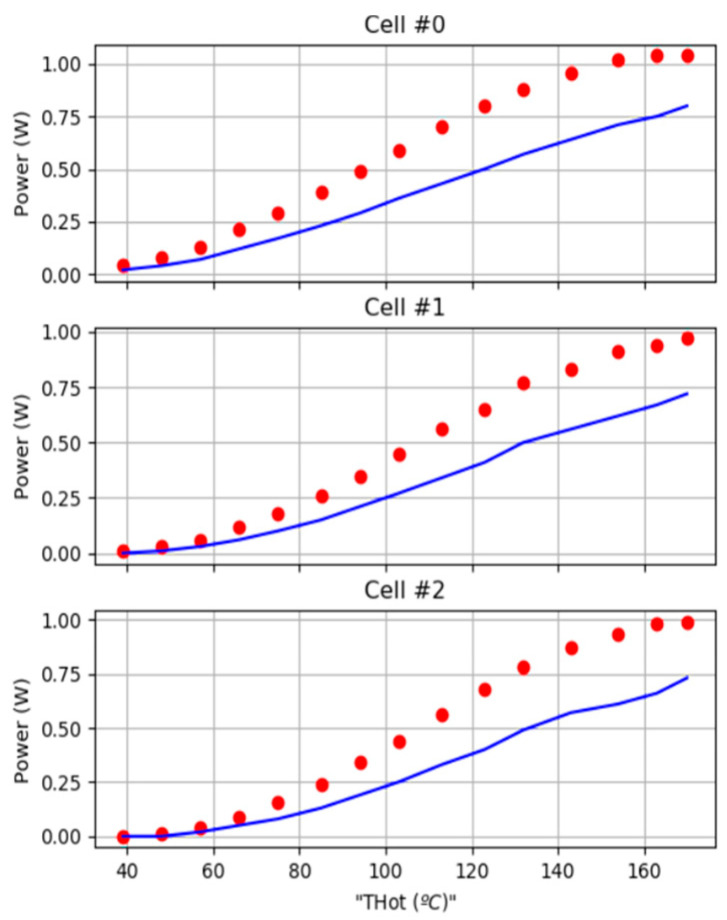
Comparison of the theoretical power computation (red dots) with respect to experimental power (blue line) as a function of the hot-face and the cold-face temperatures.

Figure 12 compares the experimental power output with the theoretical predictions as a function of the hot-side and cold-side temperatures. The experimental results exhibit a lower efficiency, ranging from 10% to 28% below the theoretical values. The number of different elements that make up a system as complex as this, together with its diverse thermal behaviour, makes it difficult to determine with precision the origin of the errors, especially when it is the sum of the individual contributions to the error of each of the elements that compose it, also including the surrounding effects at the thermal level. Among the most prominent contributions are the following:

- The most difficult element to model is the conjunction of components that make up the geometry of the system. In the assembly formed between the IoT-WHRS base and the radiator, it is necessary to take into account the screws that join the system and the air gap used to thermally separate the base and the heatsink. The screws, despite being thermally isolated from the system, act as a conductive source of heat that surrounds the system;
  ○ Variations in the properties of the materials supplied by the manufacturer influence the calculation of the thermal capacities and resistances considered for the materials;
  ○ The roughness of the contacts between metal and metal and the Peltier cell are an important factor in the global error. This is a parameter that is difficult for the manufacturer to give and that must be extracted from generic material cutting tables. It influences the creation of air gaps between the contacts. Air gaps hinder thermal propagation, being an important factor in the global error;

Finally, the application of the effective parameter model over large temperature ranges can also introduce significant errors. According to H. Elarusi [25], the application of the effective material properties technique in thermoelectric analysis is complicated by (1) the temperature dependencies of the material properties and the existence of thermal and (2) electrical load resistances. As discussed in [25], a cell from a similar manufacturer shows good agreement in this analysis, with errors below 3% and with matched load resistances. Additional errors are introduced due to the fact that the load resistance is not kept constant over a large temperature range due to the non-constant internal resistance of the cell.

### 5.2. IIoT Node Power Characterisation

This subsection evaluates the power consumption of the IIoT node using the MQTT + TLS protocol for secure communication. MQTT + TLS is a lightweight publish–subscribe protocol secured with the Transport Layer Security (TLS) cryptographic protocol, which ensures communication security through x.509 authentication certificates.

**Figure 13:**
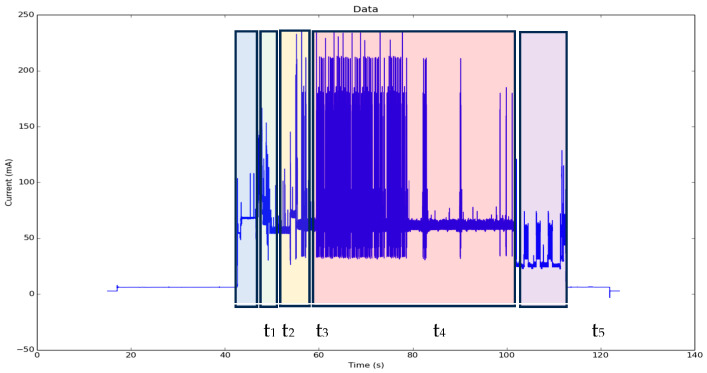
MQTT + TLS energy profile.

Two experiments were conducted to assess the energy impact of MQTT + TLS, with the results shown in Figure 13. In this figure, the major process energy consumption demands are identified:

- T_1_. Wake up;
- T_2_. Data acquisition from the three-axis vibration sensor;
- T_3_. Data processing (velocity and spectrum);
- T_4_. NB-IoT link and communication to the network;
- T_5_. Data storage for postprocessing.

Comparisons are made between the NB-IoT and LTE-CATM1 communication protocols, both implemented within the Quectel BG96 chipset, in Table 4, where we observe a significant consumption increase in LTE-CATM1 versus NB-IoT. LTE-CATM1 offers a higher speed and data capacity but at the cost of increased energy consumption due to its wider bandwidth, higher processing complexity, and greater network activity. NB-IoT, being optimised for low-power devices and IoT applications, significantly reduces its energy consumption by sacrificing speed and data capacity. This makes it ideal for applications where a low energy consumption is more important than transmission performance.

**Figure 14:**
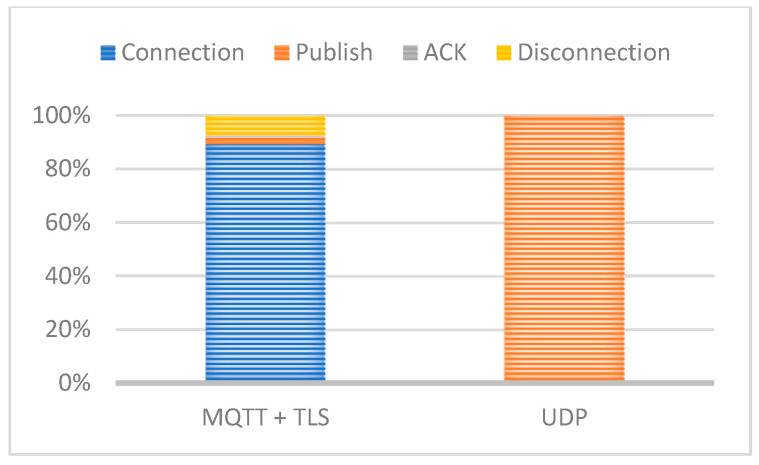
MQTT+TLS-normalised versus UDP.

Additionally, the proportion of energy consumption for MQTT + TLS compared to UDP is shown in Figure 14. The results demonstrate that MQTT + TLS, while providing robust security, incurs a higher energy cost compared to UDP due to the added cryptographic overhead.

### 5.3. Pilot Installation in a Hot Water Pump

**Figure 15:**
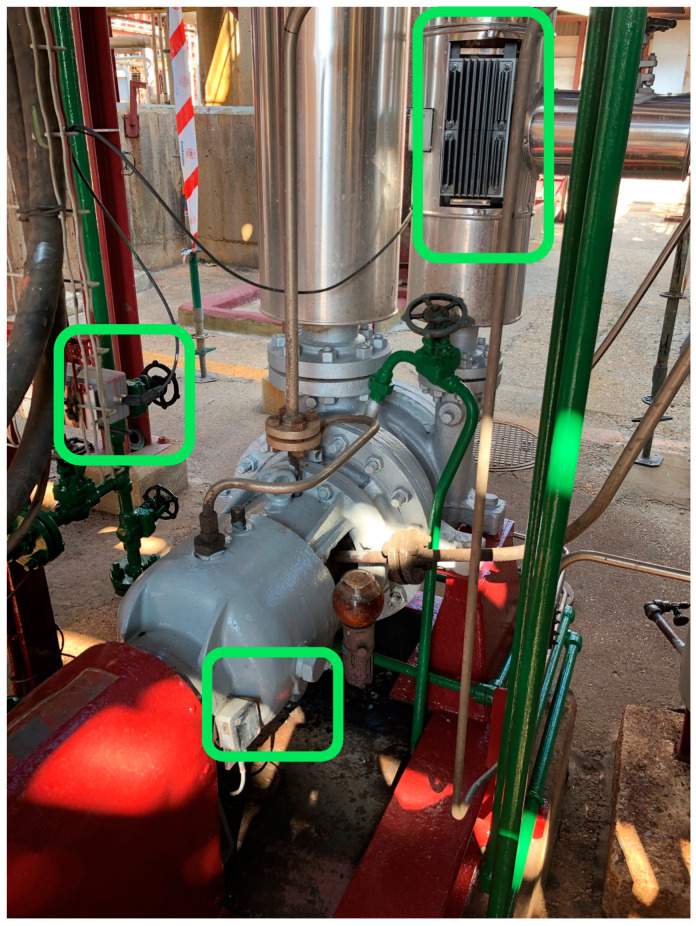
Pilot layout in CEPSA La Rábida.

This section presents the first results of the implementation. They were overseen entirely in AEInnova’s laboratory, a spin-off of the Microelectronics Department at the Autonomous University of Barcelona. Subsequently, the device will be tested under real conditions at CEPSA in La Rábida refinery in Spain, as shown in Figure 15. The complete system has been deployed to monitor vibrations in a hot water pump at CEPSA. It is easy to identify in the figure that the thermoelectric generator harvests heat from a central water pipeline (top), where the water temperature is maintained at 80 °C (data obtained from a thermographic camera). The generated energy is transferred to the NB-IoT node located on the left side of the setup, enabling data transmission. The node (middle) collects data from the three-axis vibration sensor (bottom), which is positioned at the bottom of the installation.

**Figure 16:**
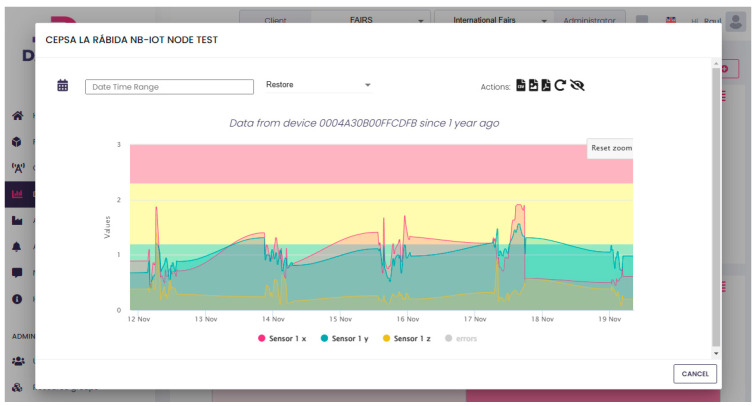
Results of 8 days of testing.

The vibration data obtained are presented in Figure 16 for a recorded time over a 8-day period. The main objective is to identify critical vibrations in this water pump.

The full process consists of converting measurements from the acceleration domain (g) to the velocity domain (RMS in mm/s). This task is performed inside the SoC processing unit. Data are presented following the ISO 10816 standard for predictive maintenance [21]. The analysis spans a frequency range of 1 Hz to 1000 Hz across three axes.

The hot water pump is classified as a Group 4 machine, with a rigid base mounted on the ground, and it must meet the ISO standard (introduced in Figure 2), which defines the following critical velocity ranges:

- Green: Healthy machine, 0 mm/s to 1.2 mm/s;
- Yellow: Short-term operation allowable, 1.2 mm/s to 2.3 mm/s;
- Red: Machine damage likely, above 2.3 mm/s.

**Figure 17:**
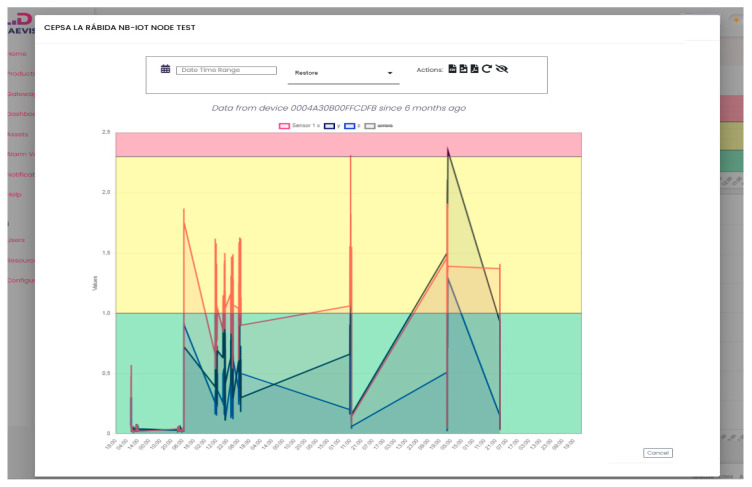
Results of 30 days of testing.

The figure highlights several periods during days 2, 3, and 4 where vibration levels significantly exceed the warning threshold of 2.3 mm/s in the Y-Axis and X-Axis. This indicates that the machine requires urgent maintenance to prevent severe or critical damage. Figure 17 illustrates several days of machine downtime due to maintenance attempts, during which the pump repeatedly failed to operate correctly after being reconnected. This process was attempted multiple times without success. Eventually, the pump was dismantled, and the NB-IoT device was disconnected.

**Figure 18:**
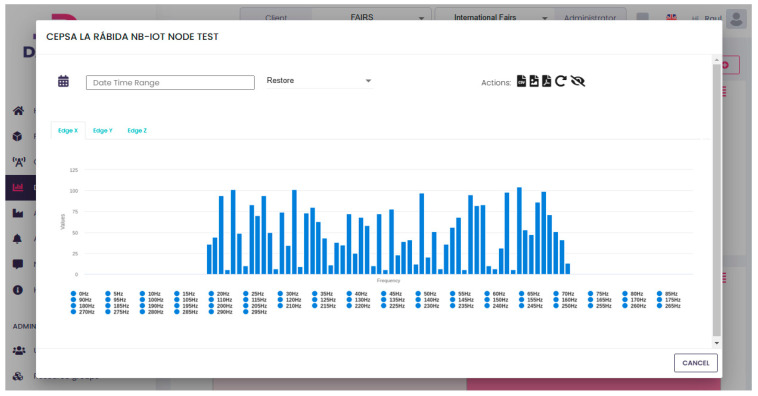
FFT of November 17th at 1.30 pm.

The spectral analysis (introduced in Figure 3) was performed thanks to the Fast Fourier Transformation (FFT). The results are shown in Figure 18. It is easy to identify that the dominant frequency components are concentrated below 300 Hz.

According to vibration diagnostic standards such as ISO 10816, this frequency range is commonly associated with mechanical faults like imbalance and misalignment. These types of faults are frequent in rotating machinery and typically produce high amplitudes at 1× or 2× the rotating speed. Although we did not have direct maintenance reports confirming the fault type in this case, the vibration signature strongly suggests such conditions. Identifying these patterns through real-time monitoring allows for early detection and targeted maintenance, thereby reducing downtime and extending the operational life of the equipment.

### 5.4. NB-IoT Connectivity Performance in a Harsh Industrial Environment

This pilot deployment was carried out within an open but highly metallic environment. The node was installed approximately 1.8 km from the nearest Vodafone NB-IoT base station, with direct line of sight. The sensor was positioned near a hot water pump and surrounded by metallic structures, including condensate return pipelines from steam traps and a steam boiler located 20 m away. Although there were no conventional walls, the dense presence of metallic elements introduced potential challenges for wireless communication, such as signal reflections and multipath interference.

Despite these conditions, the NB-IoT link remained stable throughout the pilot, enabling reliable periodic data transmission. The results suggest that NB-IoT performs adequately in such a complex industrial setting, particularly for applications with a low bandwidth and low power requirements.

### 5.5. Scalability of the IoT-WHRS Solution to Other Temperature Regimes

While the current implementation of the IoT-WHRS (Waste Heat Recovery Sensor) is optimised for medium-temperature environments (80–200 °C), the architecture can be adapted to address more extreme temperature scenarios.

In high-temperature industrial environments (above 300 °C), such as steelmaking or glass manufacturing, key design modifications would be required:

- Thermoelectric Modules: Use of materials such as silicon–germanium (SiGe) or lead telluride (PbTe), which offer better performance and stability at elevated temperatures;
- Structural Materials: Integration of ceramics or thermally robust composites to enhance insulation and mechanical durability;
- Heatsink Design: Custom heatsinks capable of handling higher thermal gradients, while maintaining an effective dissipation under constrained space and airflow conditions.

Moreover, for low-temperature or cryogenic environments, the solution would require the following:

- Thermoelectric modules optimised for small temperature differences (e.g., BiTe-based modules with enhanced thermal contact);
- Efficient thermal interfaces to minimise losses;
- Possibly even reversed thermal harvesting schemes in refrigeration cycles.

These adjustments, although not yet implemented, suggest that the proposed system architecture could be scaled or adapted to a wide range of industrial conditions with appropriate material and design choices.

## 6. Deployment Context and Industrial Considerations

### 6.1. Background and Related Work

To provide a comprehensive market analysis and assess the competitive landscape, Table 5 compares existing vibration-monitoring technologies with our proposed solution. This comparison includes leading manufacturers offering similar systems and evaluates key parameters such as technological approach, functional features, target industries, and communication protocols. Notably, our solution is the only vibration sensor on the market that utilises the NB-IoT standard for communication. Competing devices primarily rely on battery-powered systems integrated with wireless communication protocols or on wired solutions (e.g., Modbus). However, due to the energy demands of the NB-IoT protocol, battery-powered devices cannot guarantee data transmission beyond one week of operation without frequent battery replacements or recharges.

Our technology differentiates itself through its innovative energy-harvesting system, which uses residual heat as a power source instead of traditional batteries. This approach not only eliminates the limitations of finite energy sources but also aligns with sustainability goals by reducing waste and environmental impacts. Furthermore, the integration of NB-IoT for long-range wireless communication provides a significant advantage over traditional short-range systems like WirelessHART or proprietary wireless protocols, which require substantial investment in gateways and infrastructure.

By combining energy harvesting with long-range connectivity, our solution sets a new standard for industrial monitoring, offering a sustainable, cost-effective, and scalable alternative to conventional technologies.

### 6.2. Cost and Operational Reliability Considerations

The proposed batteryless IIoT sensor platform based on thermoelectric energy harvesting is designed not only for energy autonomy but also for long-term economic and operational sustainability. In terms of communication infrastructure, the system utilises the NB-IoT network provided by Vodafone in Spain. Despite transmitting vibration data every minute—including both RMS values and spectral features—the monthly data volume remains below 100 MB per node, resulting in a communication cost of less than EUR 1 per month per device. This cost efficiency makes the solution highly scalable for industrial environments with a large number of monitored assets.

Regarding long-term reliability, the thermoelectric generator (TEG) modules used in the system are based on bismuth telluride (BiTe) technology, which has demonstrated operational lifespans of 10 years or more under moderate and stable thermal conditions. In the specific use case described in this work, the sensor is installed on a hot water pump operating continuously (24/7, 365 days a year) within a refinery setting. The operating temperature is kept relatively constant, and the equipment is only shut down every three to four years for routine maintenance. Consequently, the TEG modules are not subjected to frequent thermal cycling or mechanical stress, which would otherwise degrade performance over time. This favourable scenario enhances the expected durability and reliability of the system components.

Moreover, as detailed in Section 2.1 and Figure 1, the transition from periodic to predictive maintenance can significantly reduce unscheduled downtime, maintenance labour costs, and potential collateral damage from late failure detection. Although a full Total Cost of Ownership (TCO) model is outside the scope of this study, these preliminary insights suggest that battery-free, NB-IoT-enabled vibration-monitoring systems provide a compelling trade-off between performance, cost, and maintainability in industrial environments.

## 7. Conclusions

This paper presented the modelling, testing, and application of IoT-WHRS systems designed to power IoT devices by harvesting waste heat from medium-temperature sources. These systems eliminate the need for external energy sources, enabling IoT nodes to be deployed in remote locations or critical facilities where power grids or batteries are impractical or not recommended.

The methodology proposed is generic and can be standardised for any IoT-WHRS architecture. Simulation and experimental results demonstrate strong agreement, confirming the feasibility of IoT-WHRSs as a reliable power source for IoT devices. By utilising thermoelectric technology, the system eliminates batteries, reducing maintenance costs and ensuring compatibility with explosive atmospheres (ATEX).

The integration of NB-IoT communication further enhances the system’s applicability in large industrial facilities, such as refineries, by providing long-range, low-power communication without extensive wiring. NB-IoT ensures efficient, scalable, and reliable real-time data transmission with a low latency, critical for industrial monitoring. A comparative analysis of communication protocols highlights the energy efficiency of UDP over MQTT+TLS, making it an optimal choice for IIoT applications.

Additionally, this study emphasises the benefits of predictive maintenance through vibration monitoring in both the velocity domain (RMS) and spectrum domain (Hz). This approach effectively identifies potential failures in rotating machinery, enabling timely interventions and reducing downtime.

The real-world deployment of this system at the CEPSA La Rábida refinery showcases its ability to address key industrial challenges. By combining energy harvesting, advanced communication protocols, and predictive maintenance technologies, the proposed solution offers a sustainable, cost-effective, and scalable approach to improving energy efficiency, reducing environmental impacts, and enhancing operational safety in energy-intensive industries.

## Figures and Tables

**Table 1:** IoT-WHRS materials’ properties.

	Units	Air ^(^*^)^	Al6063-T5	Graphite
Density	ρ, (kg/m^3^)	1.1839	2700	1800
Thermal conductivity	K, (W/(mK))	0.0261	209	5
Specific heat	Cp, (J/(kgK))	1004.3	896	895
Microhardness ^(^**^)^	μH, (MPa)		670	100
Surface roughness ^(^**^)^	sR, (μm)		1.8	2.0

(*) Air is treated as an ideal gas at atmospheric pressure due to its low density. The parameters are taken at T = 25 °C. (**) Microhardness and surface roughness are used in the calculation of contact resistance. The roughness of the surface depends on the method of cutting and polishing.

**Table 2:** GM200-161-12-20 Specifications and Calculated Effective Parameters.

Thermoelectric parameters		
Number of pellets, n	199	
Maximum power (Th = 200 °C), W_max_	5.3	W
Max. voltage (no load), V_max_	11.2	V
Max. current, I_max_	1.88	A
Max. efficiency, η_max_	5.6	%
Matched load resistance (200 °C), R_L_	5.9	Ω
Effective parameters		
Effective Seebeck coefficient, α*	3.33 × 10^−4^	V/K
Effective thermal conductivity, κ*	1.94	W/(m·K)
Effective resistivity, ρ*	3.8 × 10^−5^	Ω·m
Figure of merit Z*	1.6 × 10^−3^	1/K
Figure of merit ZT*	0.48	

**Table 3:** Simulation conditions and model parameters.

Simulation Setup Parameters
Rload = 10.0 Ω Torque = 0.7 N/m Force = 875,000 Pa Screw diameter = 5 mm ∅

**Table 4:** Power comparison.

	Power Consumption	
Protocol	NB-IoT	LTE-CATM1
UDP	1.17 mWh	2.15 mWh
MQTT-TLS	2.82 mWh	2.64 mWh

**Table 5:** Comparison of commercial vibration sensors.

Product/Model	Wireless Technology	Energy Source	Bandwidth	Nº Axis	Information Sent	Data Transmit
AEInnova Indueye (this paper)	NB-IOT	Waste heat.	1 Hz to 2 KHz	3	Velocity 3-axis. Spectrum.	Every 60 s up to 1 h.
Everactive/Fluke 3562 [26]	Proprietary wireless protocol. Up to 1000 nodes and 250 m.	Waste heat or solar	6 Hz a 1 KHz	3	Velocity 3-axis. Spectrum.	From 15 s to 15 min.
Emerson AMS [27]	WirelessHART. Up to 100 nodes and 50 m.	Battery. Expected 3–5 years in lab conditions.	X, Y 1 KHz, Z up to 20 KHz	3	Velocity 3-axis. Spectrum. Battery voltage.	1 h velocity, 1 time per day. Spectrum.
SKF Vibration Sensor [28]	WirelessHART. Up to 100 nodes and 50 m.	Battery. Expected 2–3 years in lab conditions.	10 Hz a 1 KHz	1	Vibration: Velocity 3-axis. Acceleration. Battery voltage. Temperature: −40 a 85 °C. Precision ± 2 °C.	Temperature every 5 min. Vibration every 1 h.
Yokogawa Sushi [29]	LoRaWAN (up to 2 Km)	Battery. Expected 2 years.	10 Hz a 1 KHz.	3	Vibration: Velocity 3-axis. Acceleration. Battery voltage. Temperature: −20 °C a 85 °C.	1 data per minute up to every 3 days.

## Data Availability

All data are available in public funding agencies: AEInnova (https://aeinnova.com/proyectos/). accessed on 18 March 2025.
